# Trajectories of Cognitive Change and Their Association with All-Cause Mortality Among Chinese Older Adults: Results from the Chinese Longitudinal Healthy Longevity Survey

**DOI:** 10.3390/bs15030365

**Published:** 2025-03-14

**Authors:** Yifang Wei, Yi Zhang, Yuansheng Li, Fanshuo Meng, Ruixiang Zhang, Zuming You, Chenxi Xie, Jiyuan Zhou

**Affiliations:** Department of Biostatistics, School of Public Health (State Key Laboratory of Multi-Organ Injury Prevention and Treatment, and Guangdong Provincial Key Laboratory of Tropical Disease Research), Southern Medical University, Guangzhou 510515, China; w12210152@i.smu.edu.cn (Y.W.); 3187042031@i.smu.edu.cn (Y.Z.); li128795@i.smu.edu.cn (Y.L.); m_22220019@i.smu.edu.cn (F.M.); zrx15751771690@i.smu.edu.cn (R.Z.); yzm886216@i.smu.edu.cn (Z.Y.); a22320064@i.smu.edu.cn (C.X.)

**Keywords:** Chinese elderly, cognitive function, growth mixture model, longitudinal trajectories, all-cause mortality

## Abstract

The analysis of cognitive trajectories is relatively underexplored in China. Furthermore, most previous studies examining the association between cognitive function and mortality have been limited to cross-sectional perspectives. This study aims to identify distinct cognitive trajectories and the corresponding influencing factors and investigate the impact of these trajectories on all-cause mortality in Chinese older adults. A total of 6232 subjects aged 65 years and above were drawn from the Chinese Longitudinal Healthy Longevity Survey. Growth mixture models were utilized to identify different cognitive trajectories, while Cox proportional hazards models were used to examine the association between the cognitive trajectories and all-cause mortality after adjusting for covariates. Four cognitive trajectories were identified: rapid decline group, slow decline group, low-level stable group, and high-level stable group. Some factors such as age, sex, and marital status were significantly associated with trajectories. Compared to the high-level stable group, adjusted hazard ratios and 95% confidence intervals (CIs) for the all-cause mortality were 3.87 (95% CI: 3.35–4.48), 1.41 (95% CI: 1.24–1.59), and 1.37 (95% CI: 1.18–1.58) for the rapid decline group, the slow decline group, and the low-level stable group, respectively, indicating that these three groups had a higher mortality risk. In summary, these findings facilitate the development of targeted health promotion measures, which have implications for reducing the social and economic burdens of cognitive decline.

## 1. Introduction

At present, population aging is a major public health problem facing the world (Humphreys, 2012). China is not only the country with the largest absolute number of the older population in the world but also one of the countries with the fastest aging populations (Liu & Sun, 2015). According to the data of the latest national population census in China, the proportion of the population aged 65 years and over has increased from 8.87% in 2010 to 13.50% in 2020 (Akimov et al., 2021). With the acceleration of population aging, it has brought many health problems, among which the decline of cognitive function has caused serious challenges to the health and the quality of life of the elderly population (Duan et al., 2020). In China, a cohort study reported an 18.80% prevalence of subjective cognitive decline among people aged 60 to 80 years (Hao et al., 2017). Cognitive decline results in a decline in daily functioning and is an important clinical feature of Alzheimer’s disease and other forms of dementia (Min, 2018; Wilson et al., 2013). Several studies showed that cognitive decline has multiple detrimental effects on physical and mental health (Clemmensen et al., 2020; Gaertner et al., 2018; S. Taylor et al., 2021), which imposes a large burden on patients, families, and healthcare systems (McGrath et al., 2020; Mehta et al., 2002).

Currently, there have been some studies focusing on the incidence and the prevalence of cognitive impairment (M. Gao et al., 2017; J. Jia et al., 2014; Matthews et al., 2013; Rocca et al., 2011) and the factors affecting cognitive function in the general population (Hsu & Bai, 2022; M. Kim & Park, 2017; Wang et al., 2022). In fact, cognitive decline is a longitudinal process. Another important area of research is to identify the trajectory of cognitive function over time in older adults and the factors influencing cognitive trajectory (P. Qiu et al., 2020). However, previous studies have shown that the differences in the cognitive trajectory of the elderly are not only reflected in the individual level but also in the group level, which means that there may be subpopulations with different trajectories over time (L. W. Li et al., 2017b; Min, 2018; Tampubolon, 2015; W. Yu et al., 2021). In addition, the influencing factors for different subpopulations may be different (P. Qiu et al., 2020). Therefore, it is of great practical significance to distinguish different cognitive trajectories over time and explore their influencing factors for understanding the complex and diverse aging process of the elderly, promoting healthy aging, and developing specific health promotion measures to reduce the disease burden associated with cognitive decline (Han et al., 2016; X. Hu et al., 2019). To date, most of the existing studies have identified two to five cognitive trajectories to differentiate individuals on decline trajectories from those on stable trajectories (Han et al., 2016; X. Hu et al., 2019; Min, 2018; Olaya et al., 2017; Park et al., 2017; P. Qiu et al., 2020; Taniguchi et al., 2017; Z. Wu et al., 2022; J. Yu et al., 2020; W. Yu et al., 2021; Zahodne et al., 2016). Notably, different trajectory models have been proposed to study this topic, of which the growth mixture models (GMMs) have become the standard statistical methods for identifying multiple unobserved subpopulations according to their longitudinal change over time, describing the longitudinal change within each subpopulation, and exploring the differences in changes among different subpopulations (Frankfurt et al., 2016; Ram & Grimm, 2009).

Most previous studies of cognitive trajectories have focused on older adults in developed countries, such as the Adult Changes in Thought study (G. Li et al., 2017a) and the English Longitudinal Study of Aging (Zaninotto et al., 2018). Although there have been reports of studies from Asian populations, such as the Japanese study (Taniguchi et al., 2017) and the Korean Longitudinal Study of Aging (Min, 2018), the analysis of this issue is still relatively less in China. Unlike Japan and South Korea, China is a low- and middle-income country, which makes its older adults different from those in other countries (Tu et al., 2020). On the other hand, the availability of the data from the Chinese Longitudinal Healthy Longevity Survey (CLHLS) has facilitated several analyses on cognitive trajectories among Chinese older adults (P. Qiu et al., 2020; Tu et al., 2020; J. Yu et al., 2020; W. Yu et al., 2021). The CLHLS was established in 1998, and the follow-up surveys were conducted in 2000, 2002, 2005, 2008–2009, 2011–2012, 2014, and 2017–2018 (Pei et al., 2022). However, to the best of our knowledge, most previous studies of cognitive trajectories based on the CLHLS cohort have only used the recruitments from one or two waves as the baseline sample, and some of these studies assumed a linear trend in cognitive function over time. For example, Qiu et al. (P. Qiu et al., 2020) only used the data of 2002 and 2005 waves to fit the linear GMM for identifying cognitive trajectories. Therefore, it is necessary to make more full use of the data of cognitive function from the CLHLS and release the assumption that cognitive function changes in a linear trend over time.

Although many studies have reported the relationship between cognitive impairment and mortality (Georgakis et al., 2016; Perna et al., 2015), most of them were conducted in high-income countries (Ablett et al., 2019; Batty et al., 2016; Connors et al., 2015; Downer et al., 2019; Gombojav et al., 2011; Griva et al., 2010; Hapca et al., 2018; Yaffe et al., 2016). In low- and middle-income countries such as China, several studies have demonstrated that cognitive impairment increases the mortality risk (An & Liu, 2016; S. Gao et al., 2014; Lv et al., 2019). However, most of the previous studies have been limited to a cross-sectional perspective, ignoring the dynamic trends in cognitive function over the course of life. Only a few studies investigated the predictive role of long-term cognitive trajectories for future mortality among the older elderly. For instance, Hu et al. (X. Hu et al., 2019) used the older adults from 1998 wave in the CLHLS as the baseline sample and found that the mortality probability trajectories followed a hierarchy roughly consistent with cognitive trajectories. So far, little is known about whether or not maintaining cognitive function stability over time may be protective against death (Marioni et al., 2014; Rajan et al., 2014). Since the CHLLS collected a rich set of longitudinal data, further exploration of the relationship between cognitive trajectories and mortality is still of interest.

In summary, the purpose of this study is to (a) make full use of the CLHLS cohort to distinguish the cognitive trajectories among Chinese older adults aged 65 years and over; (b) identify the factors influencing these cognitive trajectories; and (c) explore the impact of these cognitive trajectories on all-cause mortality. We propose the following study hypotheses: distinct cognitive trajectories exist in the Chinese elderly population; some factors are significantly associated with cognitive trajectories; and cognitive trajectories can influence all-cause mortality. As such, this study may supplement the deficiencies of previous studies on cognitive trajectories and propose new insights into the development of targeted health promotion measures.

## 2. Materials and Methods

### 2.1. Study Design and Subjects

The data used in this study were made available from the CLHLS (https://opendata.pku.edu.cn/dataset.xhtml?persistentId=doi:10.18170/DVN/WBO7LK, accessed on 27 March 2023) (Center for Healthy Aging and Development Studies, 2020). The CLHLS is a longitudinal study that aimed at investigating the health status of the elderly in China since 1998. Eight waves of interview (1998, 2000, 2002, 2005, 2008–2009, 2011–2012, 2014, and 2017–2018) were conducted from 1998 to 2018. The survey covered 23 provinces, municipalities, and autonomous regions across China. People aged 65 years and above and their adult children aged 35–64 years were surveyed (Zeng et al., 2017). Data about demographic information, socioeconomic status, lifestyle characteristics, disease status, and psychological characteristics were collected by face-to-face interviews, and the survey included a total of 113 thousand interviews, of which 67.40% were from elderly people aged 80 years and above. In each wave, the CLHLS re-interviewed approximately one-third of the older adults in the previous wave while recruiting new older adults to match those lost to follow-up according to their sex, age, and education level (P. Qiu et al., 2020). We called them Cohorts 1998, 2000, 2002, 2005, 2008–2009, 2011–2012, 2014, and 2017–2018 for easy description later.

**Figure 1:**
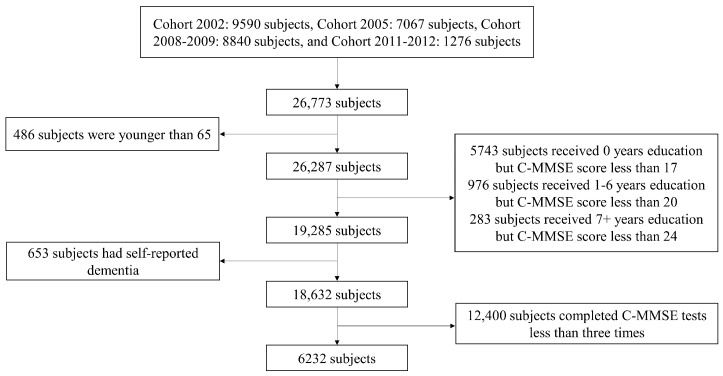
Flow chart of selecting the study sample.

In this study, we excluded the data from Cohorts 1998 and 2000, as the majority of the elderly that they included are over 80 years old, and the minimum age is 77, which may lead to inherent age bias. Note that we plan to explore the cognitive trajectories in Chinese elderly, and new recruits from Cohorts 2014 and 2017–2018 were interviewed only once or, at most, twice, which are too few for exploring the trajectories. As such, these two cohorts were also deleted. Therefore, to make full use of the CLHLS data under the condition of maintaining sufficient follow-up observations, we combined newly recruited subjects from Cohorts 2002, 2005, 2008–2009, and 2011–2012 as our study sample. To investigate the cognitive trajectories of the subjects who had no cognitive function problems, we used the following four criteria to filter the subjects (P. Qiu et al., 2020): (1) 65 years or older at the baseline; (2) the scores of the Chinese version of the Mini-Mental State Examination (C-MMSE) ≥ 17 for illiteracy, ≥20 for the subjects with 1–6 years of education and ≥24 for those with at least seven years of education at the baseline; (3) no self-reported dementia at the baseline; and (4) the completion of the C-MMSE tests at least three times. Finally, 6232 subjects whose cognitive function had been repeatedly measured 3–6 times during 2002–2018 remained in this study. A detailed flow chart of the selection process of the study sample is shown in Figure 1.

### 2.2. Cognitive Assessment

The C-MMSE was used to measure the cognitive function in the CLHLS data. It contains 24 items that belong to five dimensions: time orientation and place orientation (e.g., “What time of day is it right now?”), registration (e.g., “Table, apples, clothes, please repeat these three things.”), attention and calculation (e.g., “How much is left from continuously spending $3 from $20?”), recall (e.g., “Please repeat the three words just said.”) and language ability (e.g., “Please repeat the sentence ‘What you plant, what you will get.’”) (X. Hu et al., 2018). Except for the question “Please name as many kinds of food as possible in 1 min” (0–6 points for 0–6 answers, respectively, and 7 points for seven or more answers), the other 23 items were recorded 1 point for correct answers, and 0 points for wrong answers or no answers (Zhang, 2006). Therefore, the total score of the C-MMSE ranges from 0 to 30, with higher scores indicating better cognitive ability. Previous studies have shown that the C-MMSE is a widely used scale for measuring cognitive function in Chinese elderly (An & Liu, 2016; M. Gao et al., 2017).

### 2.3. Assessment of All-Cause Mortality

The survival status was measured by whether the subject died or survived in the survey until the last follow-up (the 2017–2018 wave), and the statuses of death and censor are encoded as 1 and 0, respectively. The survival time of a subject was calculated from the baseline (the time of the first interview) to the date of death or the date of last follow-up (see which one happened first) (W. Jia et al., 2021). The time was measured in months.

### 2.4. Covariates

The factors potentially influencing cognitive trajectories were extracted from the baseline questionnaires of the subjects, including sociodemographic characteristics, cohort information, lifestyle factors, and disease information. Sociodemographic characteristics include age (65–80 and >80 years), sex, education level (0, 1–6 and 7+ years), place of residence, economic status, and marital status. Cohort information was considered by the year in which the subject first entered the cohort (i.e., 2002, 2005, 2008–2009, or 2011–2012). Lifestyle factors (smoking, drinking, exercising, garden work, reading newspapers or books, raising domestic animals, playing cards or mahjong, watching TV or listening to radio, participating in social activities and doing physical labor regularly) were also collected. In addition, the information of diseases was collected, including hypertension, diabetes, stroke or cerebrovascular disease (CVD), and cataracts.

### 2.5. Statistical Analysis

Continuous variables were presented as mean (standard deviation (SD)), and categorical variables were presented as count (percentage) in descriptive statistics. The normal GMMs were used to find out different patterns of cognitive trajectory of the elderly in China (Ram & Grimm, 2009). The GMMs are the standard methods for identifying multiple latent subpopulations with different developmental trajectories, describing longitudinal changes in each subpopulation, and examining the differences in changes between potential subpopulations. Multiple GMMs with different trajectory shapes were considered, including linear, quadratic, and freely estimated models. The general form of the linear unconditional GMM (with no covariates) with K classes is as follows:

$$Y_{it}=\sum_{k=1}^{K}P\left(c_{i}=k\right)\left[\alpha_{ki}+\beta_{ki}\lambda_{t}+\varepsilon_{kit}\right]\;\alpha_{ki}=\mu_{\alpha_{k}}+\zeta_{\alpha_{ki}}\;\beta_{ki}=\mu_{\beta_{k}}+\zeta_{\beta_{ki}}\;i=1,2,\ldots ,6232;\;t=1,2,\ldots ,6$$

where $Y_{it}$ is the C-MMSE score of individual i at visit time t; k is the latent class (k=1,2,…,K); $c_{i}$ is the class that individual i belongs to; $P\left(c_{i}=k\right)$ is the probability that individual i belongs to class k; $\alpha_{ki}=\mu_{\alpha_{k}}+\zeta_{\alpha_{ki}}$ and $\beta_{ki}=\mu_{\beta_{k}}+\zeta_{\beta_{ki}}$ are, respectively, the intercept and the slope of class k for individual i, where $\mu_{\alpha_{k}}$ and $\mu_{\beta_{k}}$ are the mean intercept and the mean slope over time, and $\zeta_{\alpha_{ki}}$ and $\zeta_{\beta_{ki}}$ are the corresponding random errors; $\lambda_{t}$ is the factor loading at time t, which takes the values from 1 to 6; and $\varepsilon_{kit}$ is the error term of individual i at time t and $\varepsilon_{kit}\sim N(0,\sigma_{k}^{2})$, where $\sigma_{k}^{2}$ is the variance of $\varepsilon_{kit}$. Compared to the linear GMM, the cognitive trajectories in the quadratic GMM were specified as the quadratic function of the visit time with the terms $\lambda_{t}$ and $\lambda_{t}^{2}$. As for the freely estimated GMM, only the factor loadings of the first three visit times ($\lambda_{1}$, $\lambda_{2}$, and $\lambda_{3}$) were specified, while those of the other three visit times ($\lambda_{4}$, $\lambda_{5}$, and $\lambda_{6}$) were freely estimated (Meredith & Tisak, 1990), and the other settings of the freely estimated GMM that we used here are consistent with the linear GMM described above. We considered three trajectory shapes above to determine the best trajectory model over time. To find out the number of the best-fitting classes, we built the unconditional GMM of two to five classes. The following model fitting indices were adopted to evaluate the goodness of fit of the models: (1) Akaike information criterion (AIC), Bayesian information criterion (BIC), and sample-size adjusted BIC (ABIC), with the smaller values of these indices representing a better fitting model (Ram & Grimm, 2009). (2) The entropy measures the classification accuracy of the model, and a larger entropy denotes the higher classification accuracy. An entropy value greater than 0.800 indicates that the classification accuracy of the model is more than 90% (Ram & Grimm, 2009). (3) The Vuong–Lo–Mendell–Rubin likelihood ratio test (VLRT) was used to compare the K-class model with the (K−1)-class model, and a *p*-value less than 0.05 indicates that the K-class model is more suitable for the data than the (K−1)-class model (Ram & Grimm, 2009). (4) The size of the minimum class should be no less than 5% of the sample to ensure that each class in the model contains sufficient individuals (Nagin & Odgers, 2010; Ye et al., 2020b). After the optimal model was ascertained, the logistic regression model was applied to explore the impact of the covariates on different cognitive trajectories, and the odds ratio (OR) value was used to measure the effects of the covariates on the cognitive trajectories.

Cox proportional hazard model was applied to assess the effects of the trajectories of cognitive function on all-cause mortality and calculate the hazard ratio (HR) and the corresponding 95% confidence interval (CI), which quantified the effect size. Kaplan–Meier method was utilized to estimate the survival curves by cognitive trajectory classes. We established the following four models: Model 1 is the unadjusted basic model, which only includes the cognitive trajectory as the independent variable. The influence of the confounding factors was analyzed in the other three models. In Model 2, we adjusted for the sociodemographic characteristics and the cohort information of the subjects. In Model 3, we further added lifestyle-related variables. In Model 4, we further added disease information. To analyze the effect of cognitive trajectories on all-cause mortality in a specific population, we performed subgroup analyses by age, sex, education level (dichotomized to not educated and educated), and marital status, respectively.

In all the analyses, a *p*-value of less than 0.05 was considered statistically significant. The GMMs were performed in Mplus 8.0, and all the other statistical analyses were implemented in R 4.2.1.

## 3. Results

### 3.1. Characteristics of Study Sample

Table 1 shows the characteristics of the subjects at baseline. Among the 6232 subjects, the mean C-MMSE score is 27.44 (SD = 2.96). In total, 2962 (47.53%), 1319 (21.16%), 1677 (26.91%), and 274 (4.40%) subjects were included in Cohorts 2002, 2005, 2008–2009, and 2011–2012, respectively. More than 70% of the subjects were over 80 years of age, and nearly half (48.59%) were male. More than half (51.77%) of the subjects were illiterate, and only a small proportion (12.81%) of the subjects received the education of high school and above. Nearly two-thirds of the sample lived in the countryside. A small proportion (16.17%) were rich, and people of ordinary economic status accounted for nearly 70%. More than half (57.56%) of the subjects were with a spouse. More than 70% were not smoking, and more than 70% were not drinking. More than a third of the subjects exercised. The subjects who did garden work accounted for more than 20% of the total sample, and the same for the subjects who read newspapers/books and played cards/mahjong. A total of 41.58% of the subjects raised domestic animals, more than 80% watched TV/listened to radio and did physical labor regularly, and less than 20% of the subjects did social activities. Nearly 20% of the subjects suffered from hypertension, and less than 10% suffered from diabetes, stroke/CVD, and cataract. Supplementary Figure S1 depicts a spaghetti plot of the longitudinal course of C-MMSE scores of the subjects. It can be seen from the figure that the cognitive changes in the subjects are not the same, so it is meaningful to explore the cognitive trajectory of the subjects. Supplementary Table S1 shows the longitudinal measures of the C-MMSE score per visit available in Cohorts 2002, 2005, 2008–2009, and 2011–2012. We found in Supplementary Table S1 that there have been 6, 5, 4, and 3 visits for the people in Cohorts 2002, 2005, 2008–2009, and 2011–2012, respectively. The numbers of the people in the first three cohorts gradually decreased when the follow-up proceeded, while the number of the people in Cohort 2011–2012 in the first three visits remained unchanged.

### 3.2. Cognitive Trajectories

Table 2 displays the fitting indices of the GMM based on two to five classes of cognitive function. It was found that the values of the AIC, the BIC, and the ABIC gradually decreased with the increase in the number of the classes. But the sizes of the smallest classes of the five-class models and the four-class quadratic model were less than 5%, so they were excluded. For the rest of the models, we simultaneously considered the values of the AIC, the BIC, the ABIC, and the entropy to choose the optimal model. The four-class freely estimated model was identified as the best-fitting model to represent the cognitive trajectory (which is highlighted in bold in the table) since the AIC, the BIC, and the ABIC of the four-class freely estimated model are the smallest among the rest of the models, and the entropy of the four-class freely estimated model is 0.892. Although its entropy is lower than other models, it still indicates a high classification accuracy.

**Figure 2:**
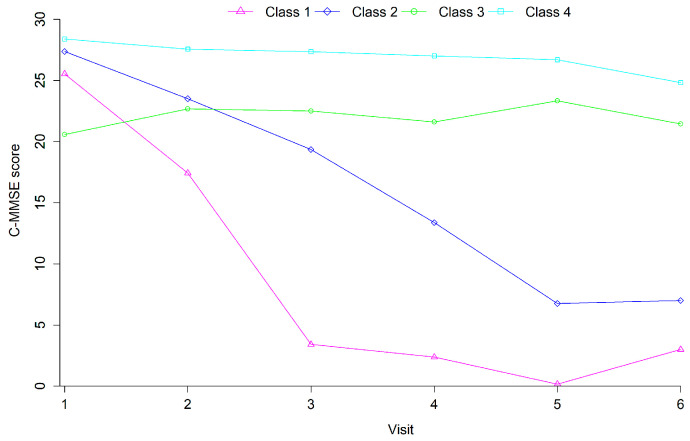
Different types of cognitive development trajectories. Note: Class 1, rapid decline group; Class 2, slow decline group; Class 3, low-level stable group; and Class 4, high-level stable group.

The changes and the trends of the four-class cognitive trajectories are shown in Figure 2. It describes the mean C-MMSE score of each class of the elderly at each visit. The characteristics of the four classes were as follows: (1) The cognitive function in the first class showed a rapid descent trend; thus, this class was named the rapid decline group (7.03%). (2) The cognitive function in the second class presented a slow descent trend, so this class was called the slow decline group (10.35%). (3) The cognitive function in the third class increased slightly throughout the whole process but maintained a relatively low C-MMSE score compared to the fourth class. Therefore, this class was labeled the low-level stable group (8.20%). (4) The cognitive function in the fourth class decreased slightly throughout the whole process but kept a higher C-MMSE score. Therefore, this class was named the high-level stable group (74.42%). Baseline characteristics of each class are shown in Table 1. From Table 1, among the four classes, the high-level stable group includes the largest proportion (83.20%) of people younger than 80 years old, the minimal proportion (43.33%) of illiteracy, and relatively less people without leisure activities. In the rapid decline group, the people without a spouse accounted for the maximal proportion (73.74%), and there were the largest proportion (14.52%) of people with cataract. Females accounted for the largest proportion (77.60%) in the low-level stable group. However, there was a larger proportion of people with stroke/CVD in the slow decline group (5.29%) and the low-level stable group (5.13%).

### 3.3. Impact of Covariates on Cognitive Trajectory Classes

The results of the multinomial logistic regression analysis on the cognitive trajectory classes are shown in Table 3. Compared to the high-level stable group, the subjects in the rapid decline group were more likely to be those from Cohort 2002 (compared to Cohort 2011–2012), older (>80 years), receiving less education, without a spouse, not doing garden work, not raising domestic animals, not playing cards or mahjong, not watching TV or listening to the radio, not participating in social activities, and with cataract; the subjects in the slow decline group were more likely to be those from Cohort 2002 (compared to Cohorts 2008–2009 and 2011–2012), older (>80 years), female, less educated, without a spouse, not raising domestic animals, and with stroke/CVD; the subjects in the low-level stable group were more likely to be from Cohort 2002 (compared to Cohort 2011–2012), older (>80 years), female, less educated, having poor economic status, without a spouse, not reading newspapers/books, not playing cards or mahjong, not watching TV or listening to the radio, not participating in social activities, and with stroke/CVD.

### 3.4. Effects of Cognitive Trajectory on All-Cause Mortality

**Figure 3:**
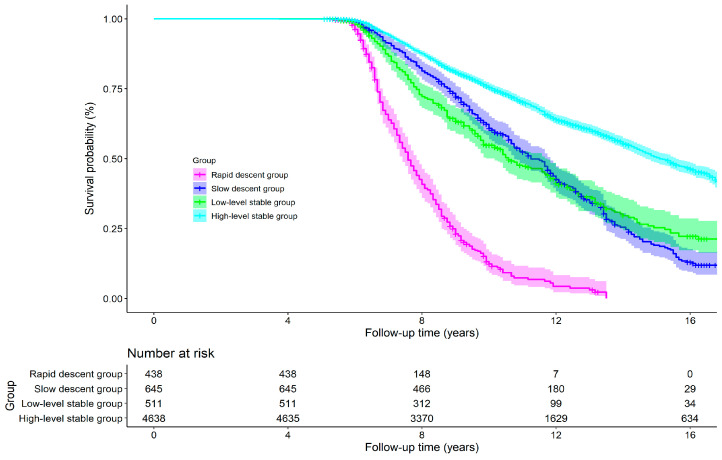
Kaplan–Meier survival curves of four cognitive trajectory groups.

A total of 2481 (39.81%) deaths were identified during the follow-up period from 2002 to 2018. Figure 3 shows the survival curves of the four cognitive trajectories. It can be seen that the median survival times are 7.58, 11.25, 10.58, and 15.08 years for the rapid decline group, the slow decline group, the low-level stable group and the high-level stable group, respectively. The influence of the cognitive trajectory on the mortality risk was analyzed according to Models 1–4, and the corresponding results are shown in Table 4. From the results of the final adjusted model (i.e., Model 4), compared to the high-level stable group, the other three groups had a higher mortality risk. The adjusted HRs and the 95% CIs for the rapid decline group, the slow decline group, and the low-level stable group are 3.87 (95% CI: 3.35–4.48), 1.41 (95% CI: 1.24–1.59), and 1.37 (95% CI: 1.18–1.58), respectively. The results of the subgroup analyses based on Models 1–4 are given in Supplementary Table S2. From the final adjusted model (i.e., Model 4), the above effects were observed in the subgroup aged 65–80 years with the adjusted HRs of 8.23 (95% CI: 6.45–10.50), 1.93 (95% CI: 1.65–2.27), and 1.50 (95% CI: 1.20–1.88), respectively. In the subgroup older than 80 years, compared to the high-level stable group, the subjects in the rapid decline group had a higher mortality risk (adjusted HR: 2.43; 95% CI: 2.04–2.90), but no statistically significant difference was found in the slow decline group (*p* = 0.138) or in the low-level stable group (*p* = 0.652). The effect size in the subgroup aged under 80 years is larger than that in the subgroup older than 80 years. In the female subgroup, the adjusted HRs are 3.92 (95% CI: 3.23–4.77), 1.48 (95% CI: 1.25–1.75), and 1.57 (95% CI: 1.31–1.89) for the rapid decline group, the slow decline group, and the low-level stable group, respectively, compared to the high-level stable group. In the male subgroup, the results of the rapid decline group and the slow decline group are similar to the female subgroup, while there is no statistically significant difference between the low and high-level stable groups (*p* = 0.518). In the population not educated, compared to the high-level stable group, the adjusted HRs and the 95% CIs for the rapid decline group, the slow decline group, and the low-level stable group are 3.55 (95% CI: 2.98–4.24), 1.29 (95% CI: 1.10–1.50), and 1.36 (95% CI: 1.15–1.59), respectively. In the educated population, compared to the high-level stable group, people in the rapid decline group (adjusted HR: 5.00; 95% CI: 3.88–6.44) and the slow decline group (adjusted HR: 1.65; 95% CI: 1.34–2.02) had higher mortality risk. However, this issue cannot be observed between the low and high-level stable groups (*p* = 0.198). In the population without a spouse, compared to the high-level stable group, the adjusted HRs and the 95% CIs for the rapid decline group, the slow decline group, and the low-level stable group are 3.49 (95% CI: 2.92–4.17), 1.29 (95% CI: 1.09–1.52), and 1.24 (95% CI: 1.03–1.50), respectively. The corresponding results in the population with a spouse are 4.71 (95% CI: 3.62–6.12), 1.55 (95% CI: 1.29–1.87), and 1.62 (95% CI: 1.27–2.07), respectively.

## 4. Discussion

In this study, based on a large nationally representative Chinese sample, we applied the GMM to explore the developmental trajectories of cognitive function in elderly individuals. We identified four distinct cognitive trajectories: rapid decline group, slow decline group, low-level stable group and high-level stable group. Some factors were found to be related to these trajectories. The subjects who were from the early cohort, older, female, less educated, without a spouse, having poor economic status, not doing garden work, not raising domestic animals, not reading newspapers/books, not playing cards or mahjong, not watching TV or listening to the radio, not participating in social activities, and suffering from stroke/CVD or cataracts were at greater risk of being classified into the cognitively poorer group. We also studied the relationship between these cognitive trajectories and all-cause mortality and found that, compared to the high-level stable group, in the case of controlling the confounding factors, the rapid decline group, the slow decline group, and the low-level stable group had a higher mortality risk.

Our results are partly consistent with those of previous studies (Min, 2018; Taniguchi et al., 2017; Tu et al., 2020), which identified stable trajectories and declining cognitive trajectories. The group of the high-level stable cognitive trajectories encompassed the majority of the subjects, suggesting that most older adults maintain relatively stable cognitive function during aging. Only a small number of elderly people belong to the cognitive decline group, especially the rapid decline group. However, similar to previous studies (Min, 2018; Zaninotto et al., 2018), these proportions are mainly dependent on the factor of age. From our results, the older the age, the more likely it is to be classified into the worse cognitive group compared to the high-level stable group. With age, the physical function of the elderly gradually deteriorates, and the cognitive function is often poor (Yang et al., 2016). Other findings also confirmed that age is a risk factor for cognitive decline (P. Qiu et al., 2020; C. A. Taylor et al., 2018). This study incorporated the cohort as a covariate into the models and found that the subjects in the later cohorts (2005 vs. 2002, 2008–2009 vs. 2002, and 2011–2012 vs. 2002) were more likely to maintain a high and stable level of cognition. This may reflect the positive effect of social progress on health, because the subjects who entered the survey later had better socioeconomic conditions, better nutritional intake in embryo and early childhood, and higher health reserves, from the perspective of life course. According to the cumulative advantage theory (Crystal & Shea, 1990), this cumulative advantage of early life could help them reduce the risk of cognitive impairment in old age. In the previous study (Lv et al., 2019), a similar finding was observed, i.e., compared to Cohort 2002, people with cognitive improvement in Cohort 2008 accounted for a higher proportion. However, it had not considered the cohort as the covariate and only compared the subjects from Cohort 2002 with those from Cohort 2008.

We observed in this study that sex is associated with cognitive trajectories, with males more likely to belong to the high-level stable group than females. This shows that, in old age, men were more conducive to maintaining high and stable cognitive function than women, which has been supported by previous studies (X. Hu et al., 2019; J. Yu et al., 2020). This may be due to several reasons, as follows: Firstly, females are at higher risk than males for occurring Alzheimer’s disease lesions and experience more severe cognitive decline (Buckley et al., 2018; R. Li & Singh, 2014). Secondly, due to patriarchal traditional social thought of China in the past, in early life, women would live with more difficulty than men and might suffer from more serious malnutrition; thus their cognitive function was severely affected (Zhang et al., 2008). Finally, under the traditional gender division of labor, women were more likely to encounter the disadvantages of unequal status in some aspects. The education level and the economic income of female elderly were generally lower. The unequal status of women in terms of socioeconomic resources made it difficult to use health services, thereby affecting their cognitive level (Song & Bian, 2014; Zhou et al., 2006). However, this sex difference was not statistically significant in the rapid decline group versus the high-level stable group, which may indicate more complex influencing factors in this sample and may require further research. Consistent with the consensus that education plays an important role in cognition (Beydoun et al., 2014; Tu et al., 2020), our results suggested that people with higher levels of education are more likely to belong to the high-level stable group. It has been widely recognized that higher education is beneficial in slowing the decline of cognitive function in the elderly (Beller et al., 2022; Gardeniers et al., 2022; X. Hu et al., 2019; Taniguchi et al., 2019; Yaffe et al., 2010).

We found that good economic status in older adults was associated with better cognitive function in the initial phase. Similarly, a previous study exploring the heterogeneous health status of older adults had also shown that older adults with higher incomes are more likely to have better health status (Ye et al., 2020a). Our finding that the subjects with a spouse are more likely to maintain a good and stable cognitive function, which is consistent with previous studies (M. Hu et al., 2021; Singham et al., 2021; Su & Xiao, 2022). The explanation for this is that older people with a spouse often live with their companion, maintaining certain communication activities with the companion; thus, they can receive more emotional support, which is more conducive to reducing the possibility of cognitive decline (Lee et al., 2019). Widowed or unmarried elderly lack life care and spiritual comfort from their spouses, which negatively affects their physical and mental health and increases the risk of cognitive decline. Our findings also showed that older adults who engage in leisure activities (doing garden work, reading newspapers or books, raising domestic animals, playing cards or mahjong, watching TV or listening to the radio, and participating in social activities) are less likely to experience cognitive impairment. This is similar to the previous results (Ding et al., 2022; R. X. Jia et al., 2019; D. Kim et al., 2017; Mao et al., 2020; J. Qiu et al., 2019; Su & Xiao, 2022). Recreational activities in the elderly can increase the opportunities to communicate with others, improve physical and mental health, and help the elderly maintain their cognitive functions. Therefore, older people should be more active in leisure and social activities.

We saw that the proportions of people with diseases (hypertension, diabetes, stroke/CVD and cataract) in our study sample are low. So, we examined the original sample (26,773 subjects) and found that the proportions of people suffering from these diseases in the original sample are 17.15%, 2.43%, 5.37%, and 11.65%, respectively, which are similar to our study sample. Growing evidence linked chronic diseases to cognitive impairment in older adults (Gardeniers et al., 2022; Lee & Cho, 2021; Lin et al., 2022; Yohannes et al., 2017). Our results also demonstrated that stroke/CVD and cataracts lead to functional decline. Previous studies have found that stroke was associated with cognitive impairment and dementia in the elderly (Kuzma et al., 2018; Rajan et al., 2014; Savva & Stephan, 2010), and it has been reported that stroke increased the risk of cognitive impairment by at least 5–8 times (Kulesh et al., 2018). For CVD, a review (Zimmerman et al., 2021) in 2021 mentioned that one of the key factors for improving cognitive function in the elderly through a healthy and good lifestyle is a healthy vascular system. In addition, substantial evidence documented the association between CVD and cognitive impairment (Arvanitakis et al., 2016; Fu et al., 2004; Roher et al., 2011; Wendell et al., 2012; White et al., 2002). There was usually an intuitive explanation for this, i.e., the normal performance of brain function and the maintenance of high brain metabolic levels require stable and sufficient cerebral blood flow, and the autoregulation function of cerebral blood flow is one of the important factors to maintain cerebral blood flow. CVD can affect the autoregulation function of cerebral blood flow, thereby impacting cognitive function, so the healthy brain system is very important for maintaining cognitive function. To sum up, elderly people with CVD were at risk of declining cognitive function. In addition, existing studies have shown that there is a correlation between cataracts and cognitive decline (Deng et al., 2023; Tai et al., 2022), and cataract surgery can improve the cognitive function in dementia patients (Jefferis et al., 2011; Jonas et al., 2018; Maharani et al., 2018), but the specific internal explanation needs further research.

There have been limited previous studies about the effect of cognitive trajectories on subsequent mortality risk. Studies have found an increased mortality risk among people with greater overall cognitive decline (X. Hu et al., 2019; J. H. Kim & Kim, 2019; Taniguchi et al., 2019; Z. Wu et al., 2022; Yaffe et al., 2016). Our findings are consistent with previous studies that the subjects in poorer cognitive trajectories had a higher mortality risk. The mechanism by which cognitive impairment is associated with the increased mortality risk is still unclear, but the following possible mechanisms are currently considered: Firstly, cognitive decline is an early manifestation of dementia and cognitive impairment, and previous reviews have shown that patients with dementia and cognitive impairment are at an increased mortality risk (Dewey & Saz, 2001; Guehne et al., 2006). Secondly, cognitive impairment may be due to the poor control of some chronic diseases (such as stroke, CVD, etc.) by patients or due to the side effects of drugs (Batterham et al., 2012; Ruxton et al., 2015; Shipley et al., 2007). Thirdly, cognitive impairment may reflect “terminal decline”. An existing study has claimed that, when a person is close to death, the overall cognitive function will decline (Muniz-Terrera et al., 2011). Fourthly, because the skills required to acquire medical knowledge and comprehensive health information are directly related to cognitive function, people with cognitive deficit may have difficulty in recognizing the symptoms of the disease and cannot receive diagnosis and treatment earlier, resulting in worsening of the disease and shortening of life expectancy (Smith et al., 2018).

In the subgroup analyses, we found that cognitive trajectories were more strongly associated with all-cause mortality among relatively younger subjects (65–80 years vs. >80 years). The existing studies also supported a stronger association between cognitive impairment and all-cause mortality in younger older adults than in old elderly adults (Lavery et al., 2009; Lv et al., 2019; Schupf et al., 2005). Normally, younger older people have more complete and resilient cognitive abilities, and they may be less likely to experience the consequences of cognitive impairment than old elderly adults due to mild diseases. Therefore, cognitive decline in the younger older group is more of concern, which may reflect underlying brain diseases or some underlying process associated with death (Lavery et al., 2009; Lv et al., 2019).

The above findings have practical significance. Firstly, because the results of this study found that most older adults belonged to the high-level stable group, i.e., they can maintain cognitive stability, this finding favors the advancing of healthy aging. Secondly, strengthening the construction of education for the elderly and cultivating the interest of the elderly in reading books and newspapers frequently to activate their thinking will help improve cognitive function and reduce the risk of cognitive decline in the elderly. Thirdly, advocating spouse ownership among older adults and supporting remarriage among widowed older adults will reduce the risk of cognitive decline. Fourthly, according to the World Health Organization Guidelines on risk reduction in cognitive decline and dementia issued in 2019 (WHO, 2019), older people should be more involved in leisure activities, which is good for cognitive health. It is recommended to develop community places for leisure and entertainment activities, encourage the elderly people to engage in leisure activities, and create a rich environment for contacting with the outside world, which will prevent the decline of cognitive function. Finally, the elderly should maintain a healthy body and avoid chronic diseases as much as possible. Actively intervening in cataract of the elderly, improving the lifestyle of the elderly and controlling the occurrence of CVD will help the elderly to stay away from cognitive decline. Furthermore, good and stable cognitive function in old age can also reduce the mortality risk.

For the strengths of this study, we analyzed the data from the CLHLS, which was a large and nationally representative longitudinal study, and we made full use of the data, which ensured the generalizability of our findings and increased the scientific rigor. Further, we employed the method of the GMM, which is the extension of the latent class growth model, and we considered different trajectory shapes, which make the characterization of cognitive trajectory more accurate. Moreover, we included the cohort information as a covariate in the models for exploring the influencing factors of cognitive trajectory and found that the subjects in the later cohorts were more likely to maintain a high and stable level of cognition, which may reflect social progress. In addition, we also analyzed the association between cognitive trajectories and all-cause mortality, which may provide some evidence for a link between the dynamic processes of cognitive change and death. However, the following limitations of this study should be noted. First of all, cognitive function was measured by the C-MMSE, but, because cognitive function includes multiple aspects, C-MMSE may not capture all aspects of cognition like perception, processing speed, etc., and it may limit the interpretation of cognitive trajectories (Arevalo-Rodriguez et al., 2021; W. Yu et al., 2021). Secondly, all the covariates only used the baseline data, which are static data, while changes in disease statuses and leisure activities over time may be associated with changes in cognitive function, which need better consideration in the future (Olaya et al., 2017). Thirdly, items such as leisure activities were self-reported, which may limit the accuracy of estimations due to recall bias and measurement bias. Fourthly, the CLHLS was conducted with a selection bias towards the older elderly and the healthier people. Finally, due to the limitation of the data, we cannot conduct analyses of cause-specific mortality.

## 5. Conclusions

In summary, we identified four distinct cognitive trajectories and explored their influencing factors, and the associations between specific cognitive trajectories and all-cause mortality were examined. The findings in this study provided some evidence for the heterogeneity of cognitive aging, the factors influencing the cognitive trajectory in old age, and the association between cognitive trajectory and mortality risk. Future research can use other cognitive function assessment tools such as the Montreal Cognitive Assessment Scale (MoCA) (Nasreddine et al., 2005) and introduce more influencing factors, including time-varying influencing factors, which allow for more adequate evaluation and comparison to develop more targeted intervention strategies to prevent cognitive decline in older adults.

## Figures and Tables

**Table 1:** Baseline characteristics of study subjects for different cognitive trajectory classes.

Characteristic	Total Sample (n = 6232)	Class 1 (n = 438)	Class 2 (n = 645)	Class 3 (n = 511)	Class 4 (n = 4638)
C-MMSE score, mean ± SD	27.44 ± 2.96	25.53 ± 3.56	27.36 ± 2.32	20.58 ± 2.18	28.39 ± 1.70
Cohort					
2002, No. (%)	2962 (47.53)	143 (32.65)	314 (48.68)	206 (40.31)	2299 (49.57)
2005, No. (%)	1319 (21.16)	109 (24.89)	154 (23.88)	85 (16.64)	971 (20.94)
2008–2009, No. (%)	1677 (26.91)	175 (39.95)	155 (24.03)	202 (39.53)	1145 (24.69)
2011–2012, No. (%)	274 (4.40)	11 (2.51)	22 (3.41)	18 (3.52)	223 (4.80)
Age					
65–80, No. (%)	4511 (72.38)	107 (24.43)	325 (50.39)	220 (43.05)	3859 (83.20)
>80, No. (%)	1721 (27.62)	331 (75.57)	320 (49.61)	291 (56.95)	779 (16.80)
Sex					
Female, No. (%)	3204 (51.41)	287 (65.53)	414 (64.19)	371 (72.60)	2132 (45.97)
Male, No. (%)	3028 (48.59)	151 (34.47)	231 (35.81)	140 (27.40)	2506 (54.03)
Education					
0 years, No. (%)	3217 (51.77)	320 (73.23)	446 (69.36)	445 (88.29)	2006 (43.33)
1–6 years, No. (%)	2201 (35.42)	94 (21.51)	156 (24.26)	59 (11.71)	1892 (40.86)
7+ years, No. (%)	796 (12.81)	23 (5.26)	41 (6.38)	0 (0.00)	732 (15.81)
Place of residence					
City, No. (%)	2220 (36.62)	164 (37.44)	207 (32.09)	136 (26.61)	1713 (36.93)
Countryside, No. (%)	4012 (64.38)	274 (62.56)	438 (67.91)	375 (73.39)	2925 (63.07)
Economic status					
Rich, No. (%)	1006 (16.17)	73 (16.67)	106 (16.43)	49 (9.59)	778 (16.81)
Ordinary, No. (%)	4322 (69.45)	287 (65.53)	417 (64.65)	336 (65.75)	3282 (70.90)
Poor, No. (%)	895 (14.38)	78 (17.80)	122 (18.92)	126 (24.66)	569 (12.29)
Marital status					
Without spouse, No. (%)	2644 (42.44)	323 (73.74)	380 (58.91)	333 (65.17)	1608 (34.69)
With spouse, No. (%)	3586 (57.56)	115 (26.26)	265 (41.09)	178 (34.83)	3028 (65.31)
Smoking					
No, No. (%)	4641 (74.48)	374 (85.39)	521 (80.78)	415 (81.37)	3331 (71.82)
Yes, No. (%)	1590 (25.52)	64 (14.61)	124 (19.22)	95 (18.63)	1307 (28.18)
Drinking					
No, No. (%)	4745 (76.16)	364 (83.11)	506 (78.45)	423 (82.78)	3452 (74.46)
Yes, No. (%)	1485 (23.84)	74 (16.89)	139 (21.55)	88 (17.22)	1184 (25.54)
Exercise					
No, No. (%)	4097 (65.79)	306 (69.86)	467 (72.52)	405 (79.26)	2919 (62.99)
Yes, No. (%)	2130 (34.21)	132 (30.14)	177 (27.48)	106 (20.74)	1715 (37.01)
Garden work					
No, No. (%)	4970 (79.75)	393 (89.73)	556 (86.20)	448 (87.67)	3573 (77.04)
Yes, No. (%)	1262 (20.25)	45 (10.27)	89 (13.80)	63 (12.33)	1065 (22.96)
Read newspapers/books					
No, No. (%)	4576 (73.43)	375 (85.62)	544 (84.34)	490 (95.89)	3167 (68.28)
Yes, No. (%)	1656 (26.57)	63 (14.38)	101 (15.66)	21 (4.11)	1471 (31.72)
Raise domestic animals					
No, No. (%)	3641 (58.42)	319 (72.83)	407 (63.10)	296 (57.93)	2619 (56.47)
Yes, No. (%)	2591 (41.58)	119 (27.17)	238 (36.90)	215 (42.07)	2019 (43.53)
Play cards/mahjong					
No, No. (%)	4731 (75.91)	378 (86.30)	528 (81.86)	465 (91.00)	3360 (72.45)
Yes, No. (%)	1501 (24.09)	60 (13.70)	117 (18.14)	46 (9.00)	1278 (27.55)
Watch TV/listen to radio					
No, No. (%)	1030 (16.53)	136 (31.05)	164 (25.43)	172 (33.66)	558 (12.03)
Yes, No. (%)	5202 (83.47)	302 (68.95)	481 (74.57)	339 (66.34)	4080 (87.97)
Social activities					
No, No. (%)	5086 (81.61)	400 (91.32)	557 (86.36)	474 (92.76)	3655 (78.81)
Yes, No. (%)	1146 (18.39)	38 (8.68)	88 (13.64)	37 (7.24)	983 (21.19)
Physical labor regularly					
No, No. (%)	944 (15.22)	66 (15.14)	80 (12.46)	61 (12.03)	737 (15.96)
Yes, No. (%)	5258 (84.78)	370 (84.86)	562 (87.54)	446 (87.97)	3880 (84.04)
Hypertension					
No, No. (%)	4982 (80.99)	355 (82.37)	516 (80.88)	404 (80.64)	3707 (80.92)
Yes, No. (%)	1169 (19.01)	76 (17.63)	122 (19.12)	97 (19.36)	874 (19.08)
Diabetes					
No, No. (%)	6008 (97.47)	428 (98.39)	627 (97.82)	497 (98.42)	4456 (97.23)
Yes, No. (%)	156 (2.53)	7 (1.61)	14 (2.18)	8 (1.58)	127 (2.77)
Stroke/CVD					
No, No. (%)	5900 (95.44)	417 (95.42)	609 (94.71)	481 (94.87)	4393 (95.60)
Yes, No. (%)	282 (4.56)	20 (4.58)	34 (5.29)	26 (5.13)	202 (4.40)
Cataract					
No, No. (%)	5698 (92.29)	371 (85.48)	573 (90.24)	458 (91.23)	4296 (93.33)
Yes, No. (%)	476 (7.71)	63 (14.52)	62 (9.76)	44 (8.77)	307 (6.67)

Note: Class 1, rapid decline group; Class 2, slow decline group; Class 3, low-level stable group; Class 4, high-level stable group. C-MMSE, Chinese version of the Mini-Mental State Examination; SD, standard deviation; and CVD, cerebrovascular disease.

**Table 2:** Fitting indices for two- to five-class GMMs.

No. of Classes	Model	AIC	BIC	ABIC	Entropy	VLRT	Class Size (%)
2	Linear	146,471	146,565	146,520	0.931	<0.001	86.78%/13.22%
	Quadratic	146,400	146,508	146,457	0.934	<0.001	86.71%/13.29%
	Freely estimated	146,411	146,526	146,471	0.931	<0.001	86.52%/13.48%
3	Linear	145,317	145,418	145,370	0.917	<0.001	78.66%/11.78%/9.56%
	Quadratic	144,937	145,071	145,008	0.923	0.018	79.43%/10.72%/9.85%
	Freely estimated	145,167	145,288	145,231	0.920	<0.001	78.72%/11.81%/9.47%
4	Linear	144,187	144,308	144,251	0.897	<0.001	74.37%/10.27%/8.41%/6.95%
	Quadratic	143,736	143,898	143,821	0.923	<0.001	77.44%/10.25%/9.42%/2.89%
	**Freely estimated**	**144,150**	**144,292**	**144,225**	**0.892**	**<0.001**	**74.42%/10.35%/8.20%/7.03%**
5	Linear	143,246	143,387	143,320	0.908	0.004	72.27%/9.90%/9.42%/4.93%/3.48%
	Quadratic	142,728	142,917	142,828	0.899	<0.001	73.68%/9.92%/7.99%/5.12%/3.29%
	Freely estimated	143,235	143,396	143,320	0.909	0.037	72.24%/9.97%/9.45%/4.96%/3.38%

Note: The best-fitting model is highlighted in bold. AIC, Akaike information criterion; BIC, Bayesian information criterion; ABIC, sample-size adjusted Bayesian information criterion; and VLRT, Vuong–Lo–Mendell–Rubin likelihood ratio test.

**Table 3:** Associations of baseline characteristics with cognitive trajectory classes (n = 6232).

Covariate	Rapid Decline Group		Slow Decline Group		Low-Level Stable Group	
	OR (95% CI)	*p*-Value	OR (95% CI)	*p*-Value	OR (95% CI)	*p*-Value
Cohort						
2002 (ref.)						
2005	1.00 (0.74, 1.35)	0.999	0.85 (0.68, 1.07)	0.175	0.74 (0.55, 1.01)	0.053
2008–2009	0.91 (0.69, 1.21)	0.515	0.54 (0.43, 0.69)	<0.001	1.09 (0.84, 1.40)	0.535
2011–2012	0.31 (0.15, 0.63)	0.001	0.39 (0.23, 0.65)	<0.001	0.45 (0.25, 0.81)	0.008
Age						
65–80 (ref.)						
>80	9.90 (7.50, 13.07)	<0.001	4.62 (3.73, 5.72)	<0.001	4.55 (3.56, 5.82)	<0.001
Sex						
Female (ref.)						
Male	0.84 (0.63, 1.12)	0.245	0.69 (0.54, 0.87)	0.002	0.72 (0.55, 0.95)	0.021
Education						
0 years (ref.)						
1–6 years	0.56 (0.41, 0.76)	<0.001	0.60 (0.47, 0.75)	<0.001	0.27 (0.19, 0.37)	<0.001
7+ years	0.51 (0.29, 0.89)	0.017	0.55 (0.36, 0.83)	0.004	NA	NA
Place of residence						
City (ref.)						
Countryside	0.92 (0.72, 1.18)	0.520	1.06 (0.86, 1.31)	0.573	1.19 (0.93, 1.52)	0.169
Economic status						
Rich (ref.)						
Ordinal	0.90 (0.66, 1.21)	0.479	0.88 (0.69, 1.12)	0.286	1.27 (0.91, 1.78)	0.163
Poor	1.13 (0.77, 1.67)	0.537	1.25 (0.91, 1.70)	0.168	1.91 (1.30, 2.81)	0.001
Marital status						
Without spouse (ref.)						
With spouse	0.50 (0.39, 0.65)	<0.001	0.74 (0.61, 0.90)	0.002	0.67 (0.54, 0.84)	0.001
Smoking						
No (ref.)						
Yes	0.89 (0.64, 1.23)	0.463	0.99 (0.77, 1.27)	0.934	1.30 (0.97, 1.75)	0.078
Drinking						
No (ref.)						
Yes	0.98 (0.72, 1.34)	0.906	1.19 (0.94, 1.50)	0.149	1.08 (0.81, 1.44)	0.607
Exercise						
No (ref.)						
Yes	1.04 (0.81, 1.34)	0.764	0.81 (0.66, 1.01)	0.057	0.79 (0.61, 1.02)	0.066
Garden work						
No (ref.)						
Yes	0.68 (0.48, 0.98)	0.038	0.82 (0.63, 1.06)	0.134	1.09 (0.80, 1.49)	0.588
Read newspapers/books						
No (ref.)						
Yes	1.01 (0.69, 1.47)	0.980	0.96 (0.72, 1.30)	0.808	0.59 (0.35, 0.97)	0.038
Raise domestic animals						
No (ref.)						
Yes	0.65 (0.51, 0.84)	0.001	0.78 (0.64, 0.95)	0.013	0.99 (0.80, 1.24)	0.939
Play cards/mahjong						
No (ref.)						
Yes	0.65 (0.47, 0.89)	0.007	0.82 (0.65, 1.03)	0.086	0.52 (0.37, 0.72)	<0.001
Watch TV/listen to radio						
No (ref.)						
Yes	0.77 (0.59, 0.99)	0.046	0.81 (0.65, 1.02)	0.070	0.67 (0.53, 0.85)	0.001
Social activities						
No (ref.)						
Yes	0.55 (0.38, 0.82)	0.003	0.92 (0.71, 1.20)	0.544	0.60 (0.41, 0.89)	0.010
Physical labor regularly						
No (ref.)						
Yes	1.11 (0.79, 1.54)	0.551	1.20 (0.90, 1.59)	0.218	0.85 (0.61, 1.19)	0.344
Hypertension						
No (ref.)						
Yes	1.03 (0.77, 1.38)	0.846	1.15 (0.91, 1.45)	0.243	1.15 (0.88, 1.51)	0.295
Diabetes						
No (ref.)						
Yes	1.00 (0.43, 2.30)	0.992	1.07 (0.58, 1.97)	0.832	1.06 (0.48, 2.34)	0.881
Stroke/CVD						
No (ref.)						
Yes	1.53 (0.91, 2.59)	0.112	1.56 (1.04, 2.33)	0.031	1.66 (1.03, 2.69)	0.037
Cataract						
No (ref.)						
Yes	1.58 (1.12, 2.21)	0.008	1.18 (0.87, 1.61)	0.294	1.15 (0.80, 1.66)	0.453

Note: The reference is the high-level stable group. HR, hazard ratio; CI, confidence interval; and CVD, cerebrovascular disease.

**Table 4:** Effect of cognitive trajectory on mortality risk.

	Model 1		Model 2		Model 3		Model 4	
	HR (95% CI)	*p*-Value	HR (95% CI)	*p*-Value	HR (95% CI)	*p*-Value	HR (95% CI)	*p*-Value
High-level stable group	1.00 (ref.)		1.00 (ref.)		1.00 (ref.)		1.00 (ref.)	
Rapid descent group	7.20 (6.35, 8.17)	<0.001	3.92 (3.40, 4.52)	<0.001	3.88 (3.36, 4.47)	<0.001	3.87 (3.35, 4.48)	<0.001
Slow descent group	2.03 (1.81, 2.27)	<0.001	1.43 (1.27, 1.62)	<0.001	1.42 (1.26, 1.61)	<0.001	1.41 (1.24, 1.59)	<0.001
Low-level stable group	2.07 (1.81, 2.36)	<0.001	1.40 (1.21, 1.61)	<0.001	1.39 (1.20, 1.60)	<0.001	1.37 (1.18, 1.58)	<0.001
Cohort								
2002 (ref.)								
2005			0.79 (0.71, 0.88)	<0.001	0.79 (0.71, 0.88)	<0.001	0.78 (0.70, 0.87)	<0.001
2008–2009			0.82 (0.73, 0.92)	<0.001	0.82 (0.73, 0.93)	0.002	0.83 (0.73, 0.94)	0.002
2011–2012			5.08 (1.54, 16.79)	0.008	3.79 (0.90, 15.96)	0.070	3.69 (0.87, 15.59)	0.076
Age								
65–80 (ref.)								
>80			3.01 (2.71, 3.35)	<0.001	2.95 (2.65, 3.28)	<0.001	2.98 (3.67, 3.32)	<0.001
Sex								
Female (ref.)								
Male			1.58 (1.44, 1.73)	<0.001	1.54 (1.39, 1.71)	<0.001	1.54 (1.39, 1.72)	<0.001
Education								
0 years (ref.)								
1–6 years			0.97 (0.88, 1.07)	0.561	0.98 (0.88, 1.09)	0.748	0.98 (0.88, 1.09)	0.750
7+ years			0.81 (0.69, 0.95)	0.009	0.83 (0.70, 0.99)	0.046	0.83 (0.69, 0.99)	0.044
Place of residence								
City (ref.)								
Countryside			1.02 (0.93, 1.11)	0.727	1.01 (0.92, 1.11)	0.776	1.00 (0.91, 1.11)	0.897
Economic status								
Rich (ref.)								
Ordinal			0.99 (0.88, 1.10)	0.789	0.98 (0.88, 1.10)	0.745	0.97 (0.86, 1.08)	0.559
Poor			1.16 (1.01, 1.33)	0.042	1.13 (0.98, 1.31)	0.086	1.10 (0.95, 1.27)	0.205
Marital status								
Without spouse (ref.)								
With spouse			0.78 (0.71, 0.85)	<0.001	0.79 (0.72, 0.87)	<0.001	0.79 (0.72, 0.87)	<0.001
Smoking								
No (ref.)								
Yes					1.08 (0.97, 1.19)	0.157	1.08 (0.98, 1.20)	0.127
Drinking								
No (ref.)								
Yes					0.98 (0.89, 1.09)	0.074	1.00 (0.91, 1.10)	0.942
Exercise								
No (ref.)								
Yes					1.00 (0.91, 1.10)	0.927	0.99 (0.90, 1.09)	0.840
Garden work								
No (ref.)								
Yes					0.95 (0.85, 1.07)	0.415	0.94 (0.84, 1.06)	0.345
Read newspapers/books								
No (ref.)								
Yes					0.99 (0.88, 1.12)	0.900	1.00 (0.88, 1.13)	0.954
Raise domestic animals								
No (ref.)								
Yes					0.94 (0.86, 1.02)	0.141	0.95 (0.87, 1.04)	0.296
Play cards/mahjong								
No (ref.)								
Yes					1.10 (0.99, 1.21)	0.073	1.09 (0.98, 1.20)	0.113
Watch TV/listen to radio								
No (ref.)								
Yes					0.86 (0.78, 0.95)	0.004	0.86 (0.77, 0.95)	0.003
Social activities								
No (ref.)								
Yes					0.93 (0.83, 1.04)	0.198	0.92 (0.82, 1.03)	0.152
Physical labor regularly								
No (ref.)								
Yes					0.99 (0.87, 1.12)	0.879	0.98 (0.87, 1.12)	0.800
Hypertension								
No (ref.)								
Yes							1.07 (0.96, 1.20)	0.224
Diabetes								
No (ref.)								
Yes							1.01 (0.73, 1.38)	0.964
Stroke/CVD								
No (ref.)								
Yes							1.42 (1.17, 1.72)	<0.001
Cataract								
No (ref.)								
Yes							1.02 (0.88, 1.19)	0.774

Note: Taking the high-level stable group as the reference. Model 1 is unadjusted for any covariate. Model 2 is adjusted for cohort, age, sex, education, place of residence, economic status, and marital status. Model 3 is further adjusted for smoking, drinking, exercising, garden work, reading newspapers or books, raising domestic animals, playing cards or mahjong, watching TV or listening to radio, participating in social activities and doing physical labor regularly. Model 4 is further adjusted for hypertension, diabetes, stroke/CVD, and cataracts. HR, hazard ratio; CI, confidence interval; and CVD, cerebrovascular disease.

## Data Availability

The data were obtained from the Chinese Longitudinal Healthy Longevity Survey (CLHLS), which is a public available database. The data can be applied for and made available at https://opendata.pku.edu.cn/dataset.xhtml?persistentId=doi:10.18170/DVN/WBO7LK, accessed on 27 March 2023.

## Supplementary Materials

The following supporting information can be downloaded at https://www.mdpi.com/article/10.3390/bs15030365/s1, Figure S1: Spaghetti plot of longitudinal course of C-MMSE scores of subjects; Table S1: Longitudinal measures of C-MMSE score per visit available in Cohorts 2002, 2005, 2008–2009, and 2011–2012; and Table S2: Effect of cognitive trajectory on mortality risk in different subgroups.
