# Supplementary material for: Trajectories of Cognitive Change and Their Association with All-Cause Mortality Among Chinese Older Adults: Results from the Chinese Longitudinal Healthy Longevity Survey
Source: Behav Sci (Basel). 2025 Mar 14;15(3):365. doi: 10.3390/bs15030365 (PMC11939546; doi:10.3390/bs15030365)
Supplement: Supplementary file 1 [file behavsci-15-00365-s001.zip › behavsci-3470797-supplementary.pdf]

Supplementary Materials

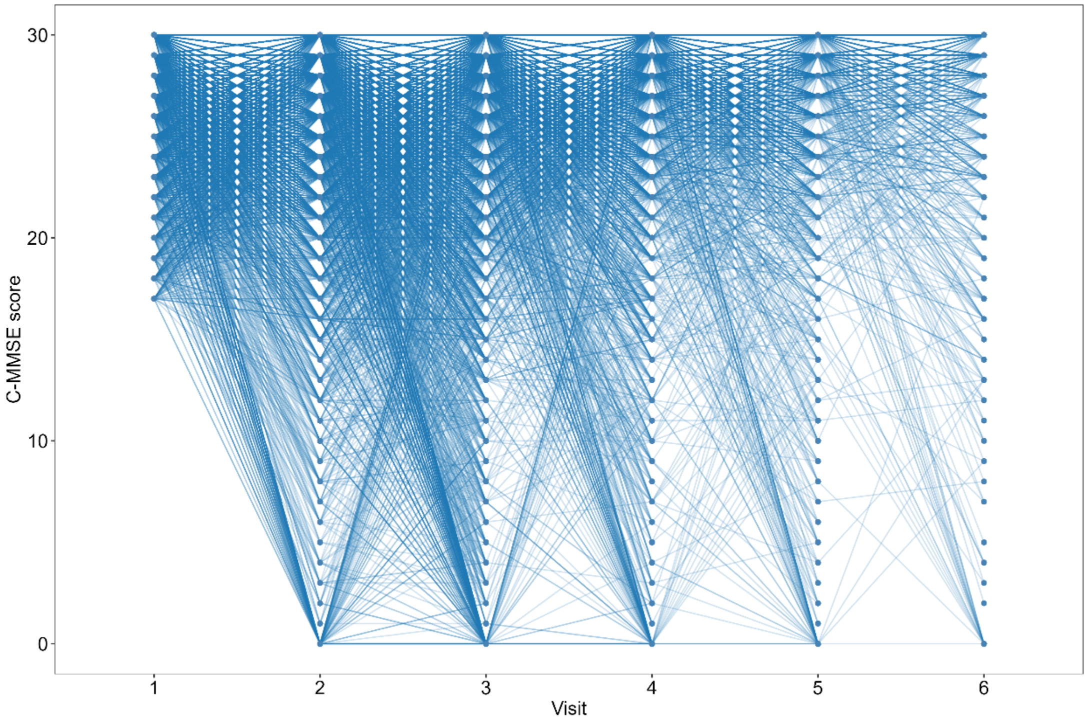

Supplementary Figure S1. Spaghetti plot of longitudinal course of C-MMSE scores of subjects.

Supplementary Table S1. Longitudinal measures of C-MMSE score per visit available in Cohorts 2002, 2005, 2008-2009 and 2011-2012

Cohort	Visit 1	Visit 2	Visit 3	Visit 4	Visit 5	Visit 6
2002	2962	2962	2957	1953	1362	635
2005	1319	1319	1301	846	427	0
2008-2009	1676	1644	1624	760	0	0
2011-2012	274	274	274	0	0	0

Supplementary Table S2. Effect of cognitive trajectory on mortality risk in different subgroups

	Model 1		Model 2		Model 3		Model 4	
	HR (95% CI)	*P*-value	HR (95% CI)	*P*-value	HR (95% CI)	*P*-value	HR (95% CI)	*P*-value
**65-80 years old**								
High level stable group	1.00 (ref.)		1.00 (ref.)		1.00 (ref.)		1.00 (ref.)	
Rapid decline group	9.25 (7.34, 11.66)	<0.001	8.61 (6.80, 10.91)	<0.001	8.33 (6.57, 10.56)	<0.001	8.23 (6.45, 10.50)	<0.001
Slow decline group	2.06 (1.77, 2.40)	<0.001	2.01 (1.72, 2.36)	<0.001	1.98 (1.69, 2.32)	<0.001	1.93 (1.65, 2.27)	<0.001
Low level stable group	1.64 (1.34, 2.02)	<0.001	1.59 (1.28, 1.98)	<0.001	1.54 (1.24, 1.92)	<0.001	1.50 (1.20, 1.88)	<0.001
**>80 years old**								
High level stable group	1.00 (ref.)		1.00 (ref.)		1.00 (ref.)		1.00 (ref.)	
Rapid decline group	2.32 (1.97, 2.72)	<0.001	2.49 (2.10, 2.96)	<0.001	2.48 (2.08, 2.94)	<0.001	2.43 (2.04, 2.90)	<0.001

Slow decline group	0.83 (0.69, 0.98)	0.031	0.88 (0.74, 1.05)	0.166	0.88 (0.73, 1.05)	0.156	0.87 (0.73, 1.05)	0.138
Low level stable group	0.98 (0.82, 1.18)	0.862	1.06 (0.87, 1.28)	0.571	1.05 (0.87, 1.28)	0.607	1.05 (0.86, 1.28)	0.652
**Female**								
High level stable group	1.00 (ref.)		1.00 (ref.)		1.00 (ref.)		1.00 (ref.)	
Rapid decline group	8.39 (7.10, 9.91)	<0.001	3.99 (3.30, 4.83)	<0.001	3.97 (3.28, 4.80)	<0.001	3.92 (3.23, 4.77)	<0.001
Slow decline group	2.40 (2.06, 2.79)	<0.001	1.49 (1.26, 1.76)	<0.001	1.49 (1.26, 1.76)	<0.001	1.48 (1.25, 1.75)	<0.001
Low level stable group	2.54 (2.15, 3.00)	<0.001	1.59 (1.33, 1.90)	<0.001	1.57 (1.31, 1.89)	<0.001	1.57 (1.31, 1.89)	<0.001
**Male**								
High level stable group	1.00 (ref.)		1.00 (ref.)		1.00 (ref.)		1.00 (ref.)	
Rapid decline group	7.31 (5.95, 8.99)	<0.001	3.88 (3.11, 4.84)	<0.001	3.88 (3.11, 4.84)	<0.001	3.93 (3.14, 4.92)	<0.001
Slow decline group	1.95 (1.63, 2.32)	<0.001	1.34 (1.12, 1.62)	0.002	1.34 (1.12, 1.62)	0.002	1.31 (1.09, 1.59)	0.005
Low level stable group	1.88 (1.48, 2.39)	<0.001	1.11 (0.86, 1.42)	0.437	1.11 (0.86, 1.43)	0.440	1.09 (0.84, 1.41)	0.518
**Not educated**								
High level stable group	1.00 (ref.)		1.00 (ref.)		1.00 (ref.)		1.00 (ref.)	
Rapid decline group	6.32 (5.41, 7.39)	<0.001	3.60 (3.03, 4.27)	<0.001	3.57 (3.01, 4.25)	<0.001	3.55 (2.98, 4.24)	<0.001
Slow decline group	1.85 (1.60, 2.13)	<0.001	1.32 (1.13, 1.53)	<0.001	1.30 (1.12, 1.52)	<0.001	1.29 (1.10, 1.50)	0.002
Low level stable group	1.96 (1.69, 2.28)	<0.001	1.38 (1.18, 1.62)	<0.001	1.37 (1.17, 1.61)	<0.001	1.36 (1.15, 1.59)	<0.001
**Educated**								
High level stable group	1.00 (ref.)		1.00 (ref.)		1.00 (ref.)		1.00 (ref.)	
Rapid decline group	9.47 (7.52, 11.94)	<0.001	4.86 (3.79, 6.23)	<0.001	4.96 (3.86, 6.37)	<0.001	5.00 (3.88, 6.44)	<0.001
Slow decline group	2.37 (1.95, 2.87)	<0.001	1.68 (1.37, 2.05)	<0.001	1.68 (1.37, 2.06)	<0.001	1.65 (1.34, 2.02)	<0.001
Low level stable group	2.10 (1.41, 3.13)	<0.001	1.33 (0.89, 1.99)	0.170	1.34 (0.89, 2.02)	0.161	1.32 (0.87, 2.00)	0.198
**Without spouse**								
High level stable group	1.00 (ref.)		1.00 (ref.)		1.00 (ref.)		1.00 (ref.)	
Rapid decline group	5.47 (4.67, 6.39)	<0.001	3.52 (2.97, 4.19)	<0.001	3.50 (2.94, 4.16)	<0.001	3.49 (2.92, 4.17)	<0.001
Slow decline group	1.72 (1.48, 2.00)	<0.001	1.31 (1.12, 1.54)	<0.001	1.30 (1.11, 1.53)	0.001	1.29 (1.09, 1.52)	0.003
Low level stable group	1.68 (1.42, 1.99)	<0.001	1.26 (1.05, 1.51)	0.013	1.25 (1.04, 1.50)	0.018	1.24 (1.03, 1.50)	0.023
**With spouse**								
High level stable group	1.00 (ref.)		1.00 (ref.)		1.00 (ref.)		1.00 (ref.)	
Rapid decline group	8.22 (6.49, 10.42)	<0.001	4.87 (3.77, 6.29)	<0.001	4.78 (3.70, 6.21)	<0.001	4.71 (3.62, 6.12)	<0.001
Slow decline group	2.02 (1.70, 2.42)	<0.001	1.55 (1.29, 1.87)	<0.001	1.57 (1.30, 1.89)	<0.001	1.55 (1.29, 1.87)	<0.001
Low level stable group	2.14 (1.71, 2.67)	<0.001	1.66 (1.31, 2.11)	<0.001	1.66 (1.30, 2.11)	<0.001	1.62 (1.27, 2.07)	<0.001

Taking the high level stable group as the reference. Model 1 was unadjusted for any covariate. Model 2 was adjusted for cohort, age, sex, education, place of residence, economic status, and marital status. Model 3 was further adjusted for smoking, drinking, exercising, garden work, reading newspapers or books, raising domestic animals, playing cards or mahjong, watching TV or listening to radio, participating in social activities and doing physical labor regularly. Model 4 was further adjusted for hypertension, diabetes, stroke/CVD, and cataracts. HR, hazard ratio; CI, confidence interval; CVD, cerebrovascular disease.
